# Cartilage-like electrostatic stiffening of responsive cryogel scaffolds

**DOI:** 10.1038/srep42948

**Published:** 2017-02-23

**Authors:** G. S. Offeddu, I. Mela, P. Jeggle, R. M. Henderson, S. K. Smoukov, M. L. Oyen

**Affiliations:** 1 Nanoscience Centre, Department of Engineering, University of Cambridge, Cambridge CB3 0FF, UK; 2 Department of Materials Science and Metallurgy, University of Cambridge, Cambridge CB3 0FS, UK; 3 Department of Pharmacology, University of Cambridge, Cambridge CB2 1PD, UK

## Abstract

Cartilage is a structural tissue with unique mechanical properties deriving from its electrically-charged porous structure. Traditional three-dimensional environments for the culture of cells fail to display the complex physical response displayed by the natural tissue. In this work, the reproduction of the charged environment found in cartilage is achieved using polyelectrolyte hydrogels based on polyvinyl alcohol and polyacrylic acid. The mechanical response and morphology of microporous physically-crosslinked cryogels are compared to those of heat-treated chemical gels made from the same polymers, as a result of pH-dependent swelling. In contrast to the heat-treated chemically-crosslinked gels, the elastic modulus of the physical cryogels was found to increase with charge activation and swelling, explained by the occurrence of electrostatic stiffening of the polymer chains at large charge densities. At the same time, the permeability of both materials to fluid flow was impaired by the presence of electric charges. This cartilage-like mechanical behavior displayed by responsive cryogels can be reproduced in other polyelectrolyte hydrogel systems to fabricate biomimetic cellular scaffolds for the repair of the tissue.

Cartilage is the biological tissue responsible for the correct functioning of the joints, covering the surface of long bones and ensuring smooth sliding and load-bearing capacity. To fulfill this role, cartilage possesses physical properties that make it a unique structural material. Its mechanical behavior depends on the presence of a large number of negative fixed charges on the aggrecan glycosaminoglycans (GAGs), polyelectrolytes comprising the extra-cellular matrix (ECM) together with collagen^1^. Up to two thirds of the elastic modulus of cartilage in compression arises from electrostatic contributions, as the hydrophilic fixed charges induce an osmotic swelling of the GAGs that is resisted by the collagen network^2^. When load is applied on cartilage, the hydrostatic pressure exerted by the fluid bound to the polyelectrolytes provides resistance against compression. Such physical behavior is not only displayed at the macroscale, but also at the scale of single cells that are adapted to the particular mechanical environment of the tissue. Synthetic cellular scaffolds intended for the repair of the tissue should therefore, possess a mechanical response tuned to that of the natural tissue^3^.

Tissue engineering scaffolds can be made to reproduce the ECM of tissues using an ever-increasing repertoire of hydrogels^4^,^5^. These are made in large part of water, which carries the nutrients required for cells to remain viable. The nanostructure of natural ECM, characterized by a network of collagen fibrils, has also inspired a range of materials, such as nanofiber-reinforced hydrogels displaying enhanced biocompatibility^6^,^7^. At the molecular level, the mechanical behavior of uncharged biological polymers has been reproduced by mimicking their network structure^8^. Yet, despite all these advances, the scaffolds produced are still often too simplistic when compared to the complex extracellular environment of natural cartilage. Instead, a biomimetic approach using polyelectrolytes for the fabrication of scaffolds may result in more biocompatible materials, as cells recognize the fixed charges on the polymers as natural cues they can interact with^9^. Synthetic charged materials could also help to better understand the electrostatic phenomena taking place in the natural tissue. Nevertheless, polyelectrolyte hydrogels are generally associated with inadequate mechanical properties, due to the increased hydrophilicity of the polymer network compared to neutral materials, and resulting plasticising of the solid phase by the interstitial fluid^9^.

Here, synthetic polyelectrolytes are used to fabricate microporous hydrogel scaffolds with enhanced mechanical behavior similar to that of natural cartilage. These responsive materials change their conformation as a result of changes in environmental pH^10^. Specifically, the carboxylic groups of polyacrylic acid (PAA) become increasingly charged when the pH is raised above their pKa (≈4), resulting in the osmotic swelling of the material^11^. PAA is blended with polyvinyl alcohol (PVA) to obtain composite hydrogels with increased mechanical stability^12^,^13^. Physical gelation through crystallization of PVA is produced by repeatedly freezing and thawing solutions of the polymer, resulting in ice templating of a microporous structure suitable for cell seeding, often called a cryogel^14^. The materials are compared mechanically and morphologically to chemically-crosslinked nanoporous gels, heat-treated in the presence of maleic acid (MA). The two types of polyelectyrolyte hydrogels are characterized as a result of fixed charge density, upon activation to the fully swollen state by a change in pH between 1 (neutral) and 13 (fully charged). The results demonstrate that electrostatic effects are fundamental in understanding and enhancing the mechanical behavior of the materials. Such results are not exclusive of the particular system here studied, but are mimetic of natural cartilage and applicable to any charged polymer system to produce functional tissue engineering scaffolds.

## Results

### Macroscale mechanical effects of charge activation

**Figure 1:**
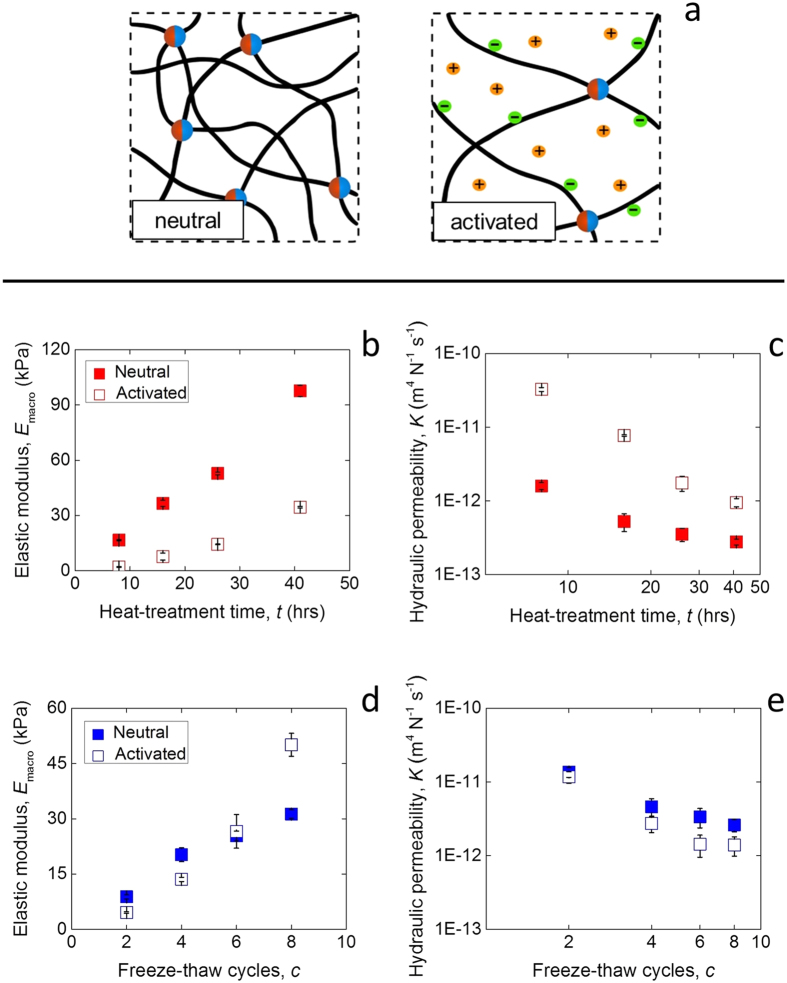
Swelling determinants of macroscale mechanical response. (**a**) Schematic of polymer network in a neutral and a charged, activated states. (**b**,**d**) Modulus and (**c**,**e**) hydraulic permeability of (**b**,**c**) heat-treated chemical hydrogels and (**d**,**e**) physical cryogels.

The macroscale mechanical behavior of the polyelectrolyte hydrogels as a function of charged state varied notably depending on the type of crosslinking (Fig. 1). The materials were mechanically tested using displacement-controlled spherical indentation. The load relaxation profiles with time were analyzed in a poroelastic framework, which considers the pressurized fluid flow away from the region of indentation contact when evaluating the elastic modulus of the materials. The heat-treated chemical gels’ elastic modulus *E*_macro_ increased with heat-treatment time *t* in both states, but was consistently smaller in the activated state. A longer heat-treatment resulted in an increased degree of crosslinking and further polymer being maintained within the network (Supplementary Fig. S1), resulting in the increased elastic modulus observed. As the network swells (Fig. 1a) the polymer volume fraction drops, resulting in a smaller elastic modulus^15^. The same considerations can be made regarding the hydraulic permeability *K* of the heat-treated chemical gels (Fig. 1c). The parameter represents the fluid mobility within the porous material, which was observed to, and is expected to decrease with increasing heat-treatment time, *i.e.* polymer volume fraction^16^,^17^. As the heat-treated gels were activated and swelled to a larger volume, their hydraulic permeability increased by up to more than one order of magnitude.

The mechanical response of physical cryogels was found to be more time-dependent than that of heat-treated chemical gels (Supplementary Fig. S2), most probably due to the presence of non-static crosslinks making the polymer network viscoelastic^18^. Yet, the response followed a similar trend to that of the heat-treated gels (Fig. 1d,e): the elastic moduli in the neutral and activated states both increased with the number of freeze-thaw cycles, *c*, known to enhance the degree of crosslinking and polymer fraction^12^. Similarly, the hydraulic permeability decreased with *c*. However, two important differences were observed upon pH-dependent swelling of the cryogels: first, the elastic modulus of the cryogels in the activated state increased faster with *c* than in the neutral state. This phenomenon resulted in the activated elastic modulus to be larger than the neutral state at large values of *c*, despite swelling. Second, the hydraulic permeability of the cryogels was consistently comparable or smaller in the activated, swollen state. Such mechanical behavior cannot be explained in terms of a decreasing polymer volume fraction with swelling, as was done with the heat-treated chemical hydrogels.

### Structural comparison of chemical and physical hydrogels

**Figure 2:**
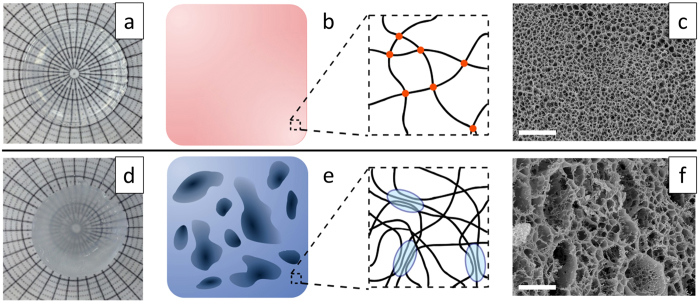
Multi-scale structure of chemical and physical hydrogels. (**a**,**d**) Photograph, (**b**,**e**) structure diagram, and (**c**,**f**) cryo-SEM micrographs (scale: 5 μm) of (**a**–**c**) chemical and (**d**–**f**) physical polyelectrolyte hydrogels.

The appearance of the chemical and physical hydrogels was compared and varied at multiple scales (Fig. 2). Upon inspection by naked eye, the heat-treated chemical gels looked entirely transparent regardless of heat-treatment time, while the physical cryogels became increasingly opaque with freeze-thawing cycles. The optical properties of the materials result from the presence of an amorphous polymer network in the chemical gels, and of light-diffracting PVA crystals in the physical cryogels^12^. The microstructure of the two types of hydrogels was observed by low temperature scanning electron microscopy (Cryo-SEM), to freeze the structure and then sublimate any surface ice before imaging. However, the process results in the disruption of the molecular networks by the growing ice crystals during freezing^19^. Although only qualitative, a difference between the two types of crosslinking could be observed clearly in the porosity (Fig. 2c,f): while the pore size of conventional heat-treated chemical gels lay in the sub-micrometer range, larger pores spanning several microns were observed in the physical cryogels due to ice-templating during crosslinking.

**Figure 3:**
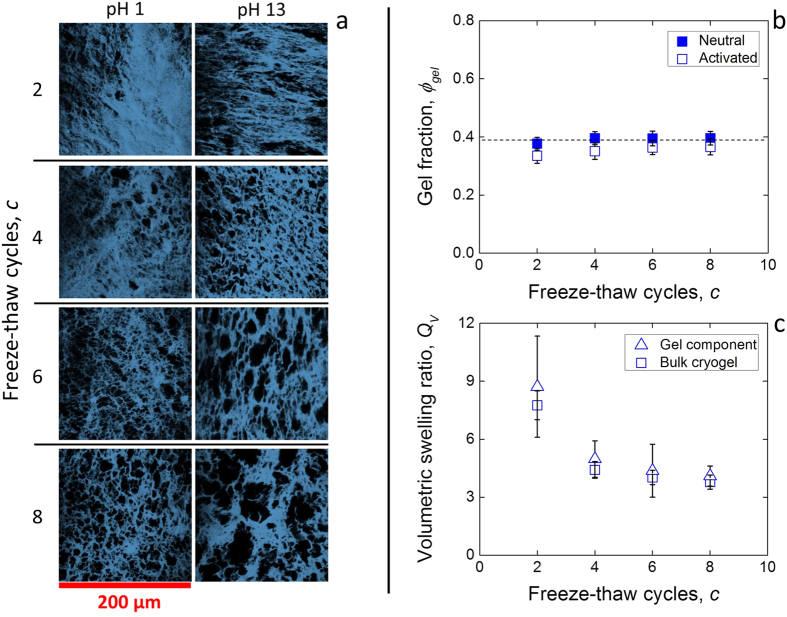
Morphology of physical cryogels. (**a**) Confocal microscopy micrographs of the cryogels as a result of swelling state and freeze-thawing cycles. (**b**) Volumetric fraction of the gel component making up the microstructure of the bulk cryogels; The dashed line is a guide for the eye. (**c**) Swelling ratio of the cryogels as a function of freeze-thawing cycles.

In an effort to understand a possible role of structure in the mechanical response of the materials, the morphology of the physical cryogels was also investigated by confocal microscopy. The technique possesses the ability to image the microporous environment of the cryogels in their hydrated state, so not to disrupt the polymer structure. Figure 3a shows the structure as a result of number of freeze-thaw cycles *c*, and activation state. As *c* increased the pore size appeared to become larger both in the neutral and activated states, with the presence of activated fixed charges resulting in consistently bigger pores. Measurements of the volume fraction of the gel component *ϕ*_gel_ from three-dimensional stacks of micrographs are displayed in Fig. 3b. This shows that although the pore structure varied between the different conditions, the gel fraction remained constant (average: 0.37, p > 0.05). A number of implications can be drawn from this morphological analysis: it appears that while repeated freeze-thawing cycles increasingly fixed the gel component into the microporous ice-templated structure, therefore increasing the pore size, the amount of such gel component did not vary. The fact that the gel fraction remained constant upon pH-dependent swelling implies that the amount of swelling of the cryogel at the macroscale is indeed determined by that of the gel component at the microscale (Fig. 3c). More importantly, this also implies that the hydrogel microstructure scales up to a larger size without densification of the material or other notable morphological change.

### Microscale mechanics of physical cryogels

**Figure 4:**
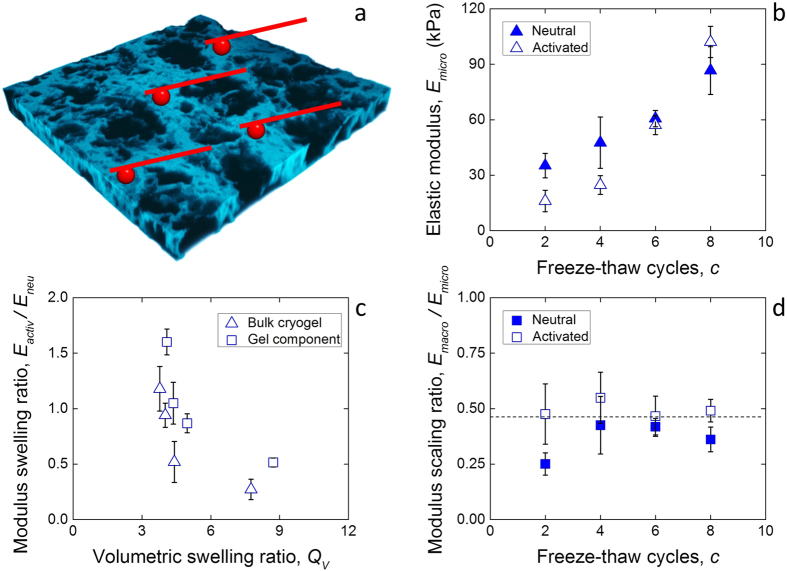
Microscale characterization of physical cryogels. (**a**) Schematic of colloidal probe AFM indentation of the cryogels microstructure. The square cryogel sample depicted has a side of 200 μm, and the tips (5 μm diameter) are shown not-in-scale for ease of visualization. (**b**) Elastic modulus of the gel component as a result of pH-dependent swelling and freeze-thawing cycles. (**c**) Ratio of macroscale to microscale moduli; The dashed line is a guide for the eye. (**d**) Relationship between the ratio of the moduli of the activated bulk cryogels and gel components and those in the neutral state, and the amount of pH-dependent swelling at the respective scale.

Cells seeded within porous tissue engineering scaffolds experience forces different from the macroscale mechanical response of the materials. In particular, the elastic modulus at the macroscale is proportional to the amount of solid within such materials, in the present case the gel component, and is a fraction of the elastic modulus of the solid component^20^. As the gel fraction in the physical cryogels does not change upon swelling, the intrinsic stiffness of the gel component was investigated and measured directly using colloidal probe atomic force microscopy (AFM) (Fig. 4a). While similar to the spherical indentation conducted at the macroscale, no time-dependent effects could be taken into consideration while testing at the microscale due to limitations of the available AFM-based indentation instrumentation. However, the shape of the indentation curves at the microscale revealed that hysteresis, and therefore time-dependence, was present in the neutral state (Supplementary Fig. S3), in which condition the elasticity was potentially over-estimated. The virtual lack of hysteresis in the activated state may have resulted from the viscoelastic crystalline crosslinks carrying less of the stress compared to the elastic polymer chains.

The elastic modulus of the gel component *E*_micro_ was consistently larger than that of the bulk cryogels (Fig. 4b compared to Fig. 1c). Also, similarly to the behavior of the materials at the macroscale, the microscale elastic modulus in the activated state also increased above the one in the neutral state after eight freeze-thawing cycles. This finding implies that the stiffening is intrinsic to the gel component, rather than a structural effect, and is translated to the cryogel at the macroscale. The ratio between the elastic moduli at the macro- and microscale (Fig. 4c) appeared to be constant after four freeze-thawing cycles (average: 0.45, p > 0.05) and comparable to the gel fraction reported in Fig. 3: a linear correspondence between elastic modulus scaling ratio *E*_micro_/*E*_micro_ and gel fraction *ϕ*_gel_ implies that stresses imposed on the structure are carried by the pore membranes^21^. After two freeze-thawing cycles the microporous structure was possibly not yet sufficiently formed for the membranes to sustain the stresses, making the structure more similar to a conventional chemical gel.

The gel component in the activated state became stiffer than in the neutral state, despite possessing a smaller polymer volume fraction (Supplementary Fig. S4). The limited extensibility of crosslinked polymer networks was shown to make hydrogels strain-stiffening by tensing the fully-extended polymer chains between crosslinks^8^. However, in the case of the present materials this effect does not appear to be responsible for the stiffening upon swelling: Fig. 4d shows that the ratio between the elastic moduli in the activated and neutral states, *E*_activ_/*E*_neutr_, decreases in magnitude as a function of volumetric swelling both at the macroscale and the microscale. A strain-stiffening hydrogel would display the opposite trend, with the modulus increasing with swelling as the network is stretched close to its ultimate capability^22^.

### Electrostatic stiffening of individual polymer chains

In order to compare the intrinsic stiffness of the polymer chains composing both the heat-treated chemical gels and the gel component of the physical cryogels, their elastic moduli were scaled over the amount of polymer present in the networks *ϕ*_p,activ_ and their degree of crosslinking 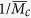, to yield the normalized modulus *E*_norm_, so that:


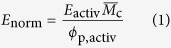


**Figure 5:**
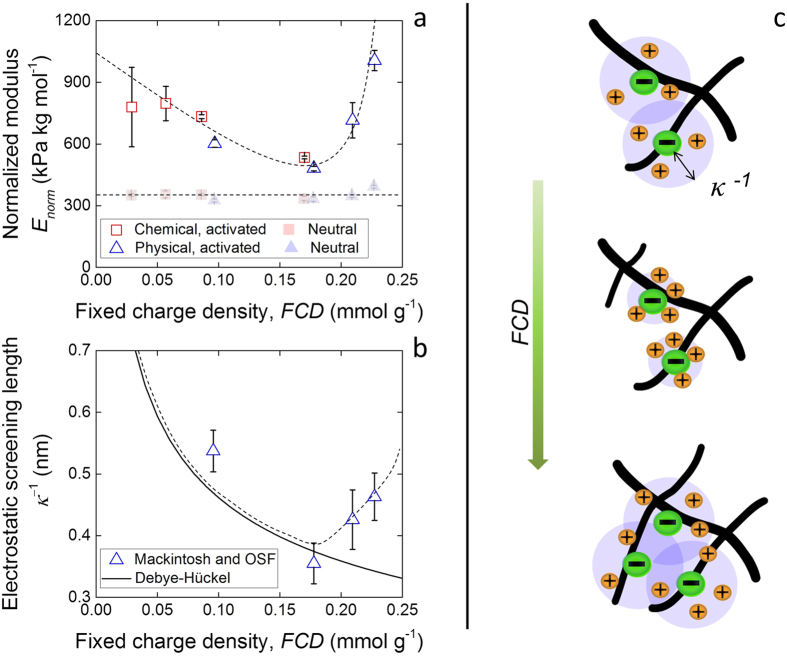
Electrostatic determinants of multi-scale mechanical response. (**a**) Normalized modulus of heat-treated chemical hydrogels and the gel component of physical cryogels as a result of fixed charge density in the activated state. The normalized modulus of the neutral gels is also shown for comparison (shaded), where the physical gel response was normalized to that of the chemical gels to take into account time-dependent effects. (**b**) Comparison between the electrostatic screening lengths expected by the Debye-Hückel theory based on the amount of fixed charges and related local ionic strength in the physical cryogels, and those calculated from the modulus of the gel component of the physical cryogels, normalized to take into account time-dependent effects. The dashed lines are guides for the eye. (**c**) Schematic of electrostatic screening length changing in size with increasing fixed charges per volume and corresponding counterions.

When the polymer volume fraction in the polyelectrolyte hydrogels increases, in particular that of PAA, so does the fixed charge density, *FCD*. The parameter was therefore calculated, assuming the mass ratio 3 : 1 of PVA to PAA is maintained in the polyelectrolyte hydrogels. Figure 5a shows the normalized elastic modulus as a function of *FCD*, as well as that of the neutral hydrogels (shaded) for comparison. The elastic modulus was observed to remain constant across degrees of crosslinking for both the chemical and physical hydrogels, although for the physical cryogels it was consistently smaller compared to the heat-treated chemical gels. This result was attributed to the presence of physical crosslinks capable of “unzipping” upon deformation, therefore decreasing the apparent magnitude of the polymer chains stiffness. Once the normalized modulus of the physical cryogels was calibrated to that of the heat-treated chemical gels, an interesting phenomenon was observed, also shown in Fig. 5a. The activated elastic moduli were larger than the neutral ones at small *FCD*. As *FCD* increased, both the elastic moduli of the chemical and physical hydrogels were observed to initially decrease, approaching the ones calculated for the neutral gels. However, as the physical crogels were able to reach larger charge densities, the elastic modulus was seen to rapidly increase with *FCD*.

The intrinsic stiffness of charged polymer chains is understood to be affected by electrostatic interactions^23^. In particular, the charges on the polymer molecules are surrounded by counterions present in solution, which effectively screen their effect to a distance given by the electrostatic screening length, *κ*^−1^. As formulated in the theory proposed by Odijk, Skolnick and Fixman (OSF), when the electrostatic effect of the fixed charges reaches further, i.e. the larger *κ*^−1^, the polymer chains get stiffer as their rotational conformation becomes constrained by charge-charge repulsion. According to the Debye-Hückel (DH) prediction, *κ*^−1^ should decrease with increasing local ionic strength^24^. In charged porous solids like cartilage, this local ionic strength is the sum of the environmental ionic strength and the *FCD*^25^. As the fixed charges become more densified, so do the counterions in solution screening their electrostatic effect. The relationship is given by:


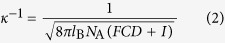


where *N*_A_ is the Avogadro’s number, the *FCD* of the chemical heat-treated gels and the gel component of the physical cryogels is expressed in mol m^−3^, and *I* is the ionic strength of the environmental solution. The Bjerrum length *l*_B_ is the distance at which the electrostatic interaction between two charges is equal to *k*_B_*T*, varying depending on the dielectric constant of the interstitial solution^24^. This length was calculated as 0.70 nm for the present system, assuming the dielectric constant of water (80.1), as it is expected that the small concentration of sodium hydroxide did not affect it significantly^26^.

The electrostatic screening length may also be directly calculated from the elastic modulus of the physical cryogels, in order to compare it to that expected by the DH paradigm, by use of the model developed by Mackintosh^8^,^27^ in conjunction with the OSF theory. The Mackintosh model takes into consideration the persistence length of the polymer *l*_p_, or the length over which the polymer behaves as a stiff rod. The shear modulus *G* of the hydrogels is then given as:


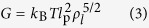


where *ρ*_l_ is the polymer density in length per volume. The shear modulus can be translated to the Young’s modulus by the relationship *E* = 2*G*(1 + *ν*), where the Poisson’s ratio *ν* of the gel component of the cryogels was assumed to be 0.5 due to the incompressibility of water. The persistence length *l*_p_ of the polymers is given by:


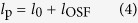


where *l*_0_ is the persistence length of the polymers in the neutral state, calculated using equation (3) from the elastic modulus of the gel component in the neutral state. *l*_OSF_ represents the increase in persistence length due to electrostatic effects, related by the OSF theory to the electrostatic screening length by ref. 24:


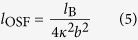


where *b* is the distance between neighboring charges, taken as the PAA monomer length (0.27 nm^28^).

The comparison between the *κ*^−1^ profile with *FCD* calculated according to the DH prediction and the calculated values from the measured elastic moduli is displayed in Fig. 5b. Once again, the results derived from mechanical testing were calibrated to the DH baseline to account for viscoelastic effects in the physically-crosslinked polymer network. The screening length could be seen to initially decrease following the DH prediction, to then abruptly increase at *FCD* larger than 0.18 mmol g^−1^. As a result, the *κ*^−1^ value reported for the largest *FCD* approached the initial one calculated for the smallest *FCD* (Fig. 5c). It thus appears that as the fixed charge density within the polyelectrolyte hydrogels increased, first the intrinsic elastic modulus of the charged polymer chains decreased to approach the one displayed in the neutral state, as expected by the OSF theory. Yet past a threshold value of *FCD*, the intrinsic elastic modulus increased again above the neutral baseline. Coupled with the larger polymer fraction, this effect can cascade up length scales, translating from the polymer chains to the gel components, to then the bulk cryogels.

## Discussion

Responsive polyelectrolyte hydrogels made from blends of PVA and PAA were mechanically characterized as a function of crosslinking mechanism. Nanoporous heat-treated chemical gels exhibited the mechanical behavior expected from traditional polymer networks mechanics^15^. On the contrary, microporous physical cyogels were found to display a different, cartilage-like response to pH-dependent swelling: in the first instance, their hydraulic permeability was seen to decrease despite the overall drop in polymer volume fraction when the pH was increased above the pKa of PAA. Morphological analysis of the cryogels showed that as swelling occurs, the volume fraction of the gel component making up the microporous structure remained constant, despite the polymer fraction within it necessarily decreasing. As the permeability to fluid flow of porous materials depends largely on the solid amount^17^, in this case the gel component, it is plausible to expect the fluid to choose the easiest flow path by traveling through the micropores, irrespective of the polymer fraction within the gel. This would result in a constant permeability with swelling. In addition to such effect, the decrease in fluid mobility reported was previously observed in cartilage as a result of a fixed charge density contributing to frictional effects between the flowing fluid and the charged solid^29^.

Crucially, the physical cryogels were found to become stiffer upon charge activation. The morphological study conducted showed that a structural effect was not responsible for the phenomenon. Instead, electrostatic effects affecting the polymer chains in the gels resulted in the stiffening: as the fixed charge density in the cryogels increased above a threshold value, the intrinsic elastic modulus of the polymer increased steeply. Such threshold value was approximately 0.18 mmol g^−1^, within the range expected for articular cartilage^2^. Interestingly, electrostatic phenomena were observed regardless of type of crosslinking. However, in the system studied the fixed charge density did not reach the threshold value in the heat-treated chemical gels. In the physical cryogels instead, the polymer was more concentrated within the microporous structure, so that the local charge density was larger.

The increase in elastic modulus was attributed to a diminished charge-screening effect of counterions in solution, resulting in larger electrostatic screening lengths. Such inflation in screening length was recently demonstrated experimentally to take place above a threshold ionic strength, when the large density of counterions forces them to interact at small distances^30^, and may be possibly caused by the formation of ion pairs in solution^31^. To the best of the authors’ knowledge, the effect was only reported in one experimental study on soft materials^32^, but no mention was made of stiffening of such materials, in that case lipid membranes. Interestingly, the authors of the study reported an ionic strength between 0.1 M and 0.2 M, *i.e.* in the range of the present study and of natural cartilage (~0.15 M 2). In addition, experiments performed on cartilage GAGs, again at an ionic strength of 0.1 M, showed that a sharp load response was recorded at a distance of several theoretical electrostatic screening lengths, where electrostatic effects should be completely shielded^33^. This suggests that electrostatic stiffening may play a larger role than previously thought in the great compressive elastic moduli of native articular cartilage.

The responsive physical cryogels studied can provide a biomimetic environment for the three-dimensional culture of cells aimed at the repair of cartilage: while chemical gels make use of often toxic crosslinkers, no crosslinking agent is required for the physical cryogels, which also provide a microporous structure for the seeding of cells. In addition, such structured cryogels are known as some of the toughest hydrogels in existence^34^, due to the better load distribution along the microstructure. Once the PAA molecular chains are fully activated, the charged environment is maintained down to physiological pH (>pKa)11. The presence of fixed charges affects the permeability of the materials to fluids, responsible for conveying nutrients to the cells seeded and determining the shear stresses imposed on these^35^. Electrostatic stiffening provides a means to enhance the mechanical response of the materials to overcome the limitations of synthetic polyelectrolytes, and could be applied to other charged hydrogel systems to obtain even greater effects. Ultimately, the synergetic effect of charge density and choice of polymer will provide unprecedented control over the bioinspired multifunctionality of cartilage tissue engineering scaffolds.

## Methods

### Materials fabrication

PVA (Mw 31,000–50,000 g/mol, 98–99% hydrolized) and PAA (Mw 450,000 g/mol) were purchased from Sigma Aldrich, UK, and used without further purification. Separate 15% wt aqueous solutions of the polymers were prepared by dissolving PVA and PAA at a temperature of 85 °C for no more than six hours under constant stirring. The solutions were left to cool and then mixed in a ratio of 3 : 1 by weight of PVA to PAA. Chemically-crosslinked gels were prepared by adding MA (Sigma Aldrich, UK) in a molar ratio of 1 : 2 to the hydroxyl groups of PVA. The solution was poured into sealed glass vials and heat-treated at 90 °C. Samples were collected after 8, 16, 26, and 41 hours. Physically-crosslinked cryogels were subjected to repeated freeze-thawing cycles, frozen for eight hours at −20 °C and then thawed for four hours at room temperature. Samples were collected after 2, 4, 6, and 8 cycles.

### Swelling, fixed charge density, and degree of crosslinking

The samples fabricated were immersed in excess solution (250 mL, minimum 14 : 1 volume ratio to that of the polyelectrolyte hydrogels) for 48 hours in either a 0.1 M HCl solution (pH 1, neutral state) or a 0.1 M NaOH solution (pH 13, activated state). The dry mass of the polymers *m*_d_, and that of the water *m*_w_ together composing the materials in the two states were found by desiccation of the samples. The polymer volume fraction was calculated as:


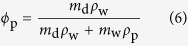


where *ρ*_w_ and *ρ*_p_ are the density of water (1 g cm^−3^) and the density of the polymers, essentially equal to 1.2 g cm^−3^ for both PVA and PAA. The fixed charge density *FCD* of the polyelectrolyte hydrogels in the activated state was calculated from first principle considerations assuming the 3 : 1 ratio of PVA to PAA is maintained after crosslinking. The volumetric swelling ratio *Q*_V_ between the neutral and activated states was calculated as:


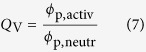


where the numerator and denominator are the polymer volume fractions in the activated and neutral states, respectively.

The polymer volume fraction in the materials after crosslinking, but before swelling in either solutions, *ϕ*_p,0_ could not be obtained with the same method due to the presence of un-crosslinked polymer within the polymer network. Instead, it was found using the measured lateral dimension of the samples before, *D*_0_, and after, *D*_neutr_, swelling to the neutral state:


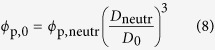


This was used to calculate the degree of crosslinking, expressed as the average molecular weight between crosslinks 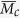, according to the well-established model by Peppas and Merrill^36^. For this, a Flory polymer-solvent interaction parameter *χ*_1_ = 0.49 was used^15^.

### Low temperature scanning electron microscopy

Cryo-SEM was performed on a Verios 460 machine (FEI, US) equipped with a cryo-transfer system (Quorum Technologies, UK). Micrographs of the frozen materials were acquired at random locations, using an accelerating voltage of 5 kV.

### Microstructure of physical cryogels

The physical cryogels were stained with Fluorescein isothiocyanate (FITC, Sigma Aldrich, UK), and their microstructure was observed on a Leica TCS SP5 system (Leica DMI 6000 B microscope, excitation: 100 mW, 488 nm Argon ion laser) and Leica LAS AF confocal software (Leica, UK). The 20 μm z-stacks were taken at 1 μm intervals. The stacks were then binarized using the BoneJ plugin in the ImageJ software distribution FIJI, which yielded the volumetric percentage of gel component *ϕ*_gel_ as the area fraction of the component over averaged over the z-direction. This was used to calculate the polymer volume fraction in the gel component by multiplying the bulk polymer amount *ϕ*_p_ by a factor 1/*ϕ*_gel_.

### Macroscale spherical indentation

Spherical indentation of the materials in both the neutral and activated states were conducted after swelling took place, and performed in the respective fluid using a glass spherical tip with a diameter of 16 mm at an indentation depth of 0.2 mm. Displacement-control mode was used on an Instron 5544 universal testing machine (Instron, US) with a ramp of ten seconds, followed by a hold of 120 seconds. A poroelastic framework of analysis, which assumes an Hertzian spherical contact^37^, was applied using an algorithm based on exponential fitting of the last (10%) section of the load relaxation profile, where this relaxation can be assumed to be due to the fluid movement only^38^,^39^. This characterization yields the elastic modulus of the materials, *E*_macro_, as well as their hydraulic permeability, *K*.

### Microscale spherical indentation

Mechanical testing of the gel component making up the microporous cryogels was performed in fluid on a Bruker Dimension Icon atomic force microscope (AFM, Bruker, US). The cryogels were loaded onto a microscope glass slide and held in place using a high density agarose bed. A 5 μm diameter glass spherical tip (sQube, Germany) was used to probe the gel down to an indentation depth in the range of 0.5 μm to 1.5 μm at a frequency of 1 Hz. The Hertz spherical contact model^37^ was applied to the approach curves to extract the modulus *E*_micro_ of the gel component. A Poisson’s ratio of 0.5 was assumed for the materials due to the expected absence of significant time-dependent effects at the frequency used for the testing. More than 50 force-displacement curves were recorded per sample. Two distributions of moduli were obtained for each sample, corresponding to the indentation of the gel component or the water-filled pore space (Supplementary Fig. S5). The latter results were readily recognized as being much smaller than those for the cryogels at the macroscale, and were therefore omitted from the analysis.

### Statistical analysis

The reported trends were tested for statistical significance using one-way analysis of variance (ANOVA). When comparison was made between different trends, paired t-tests were used. A probability value of 95% (p < 0.05) was used to determine significance for both tests. All statistical analysis was carried out using the statistics package in Origin Pro 2015 (Origin Lab, US). Values are reported as means and standard deviations, where at least two physical samples per condition were tested.

## Additional Information

**How to cite this article**: Offeddu, G. S. *et al*. Cartilage-like electrostatic stiffening of responsive cryogel scaffolds. *Sci. Rep.* **7**, 42948; doi: 10.1038/srep42948 (2017).

**Publisher's note:** Springer Nature remains neutral with regard to jurisdictional claims in published maps and institutional affiliations.

## Supplementary Material

Supplementary Information
