# Supplementary material for: Cartilage-like electrostatic stiffening of responsive cryogel scaffolds
Source: Sci Rep. 2017 Feb 23;7:42948. doi: 10.1038/srep42948 (PMC5322396; doi:10.1038/srep42948)
Supplement: Supplementary Information [file srep42948-s1.pdf]

Supplementary information for "Cartilage-like electrostatic stiffening of responsive cryogel scaffolds"

G.S. Offeddu, I. Mela, P. Jeggle, R.M. Henderson, S.K. Smoukov, M.L. Oyen

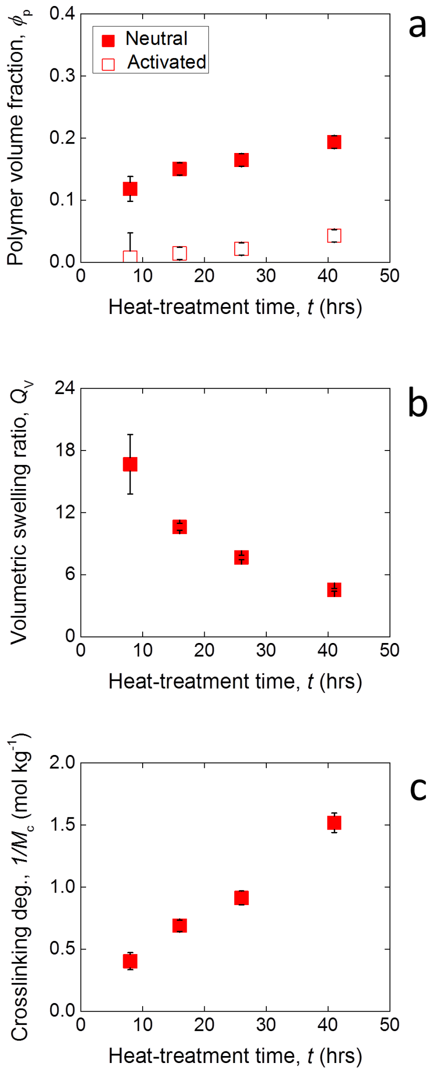

Supplementary Figure S1: Characterization of chemical hydrogels. (**a**) Polymeric volume fraction, (**b**) volumetric swelling ratio, and (**c**) degree of crosslinking of the chemical heat-treated gels as a result of heat-treatment time.

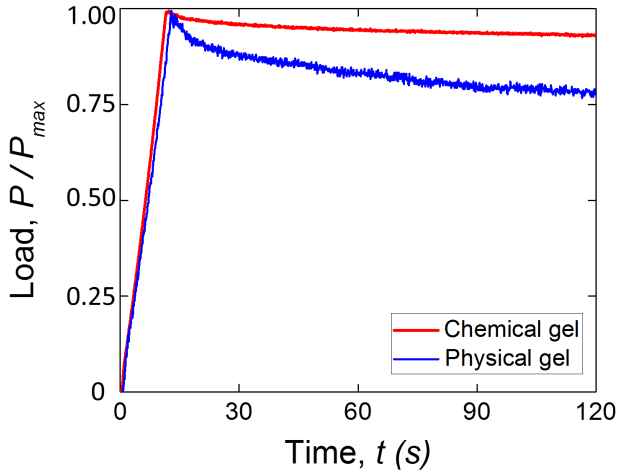

Supplementary Figure S2: Macroscale time-dependency comparison. Representative load response with time upon indentation of the chemical and physical hydrogels at the macroscale.

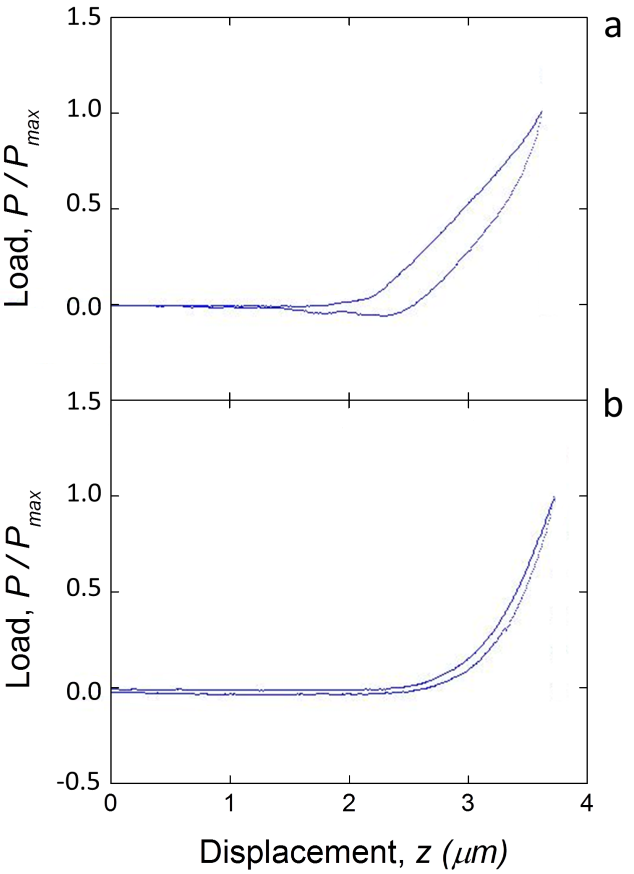

Supplementary Figure S3: Microscale load response. Colloidal probe AFM representative indentation curves for the gel component in the (**a**) neutral and (**b**) activated states.

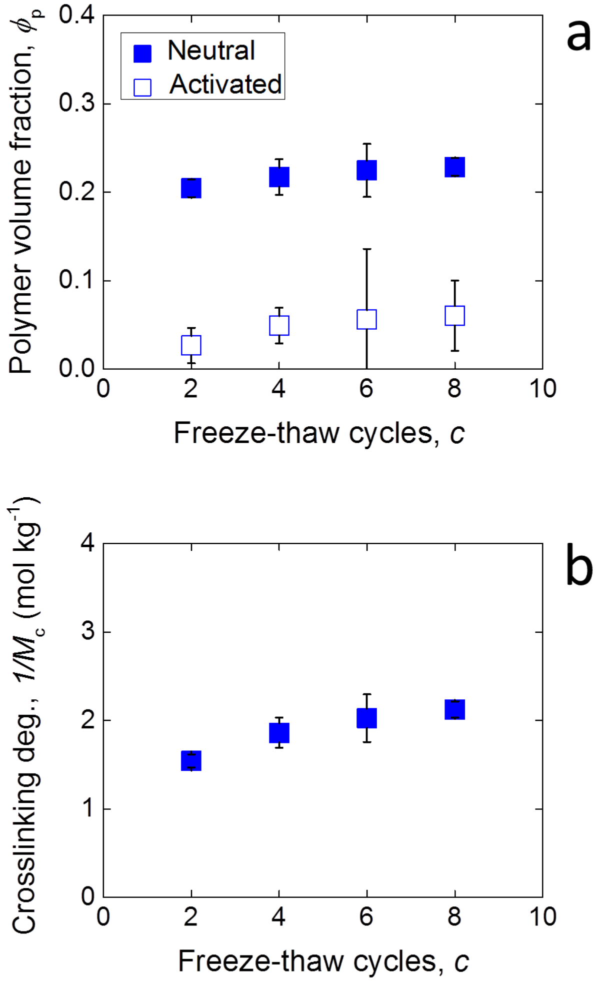

Supplementary Figure S4: Characterization of physical gel component. (**a**) Polymeric volume fraction, and (**b**) degree of crosslinking of the gel component making up the microporous physical cryogels, as a function of number of freeze-thawing cycles.

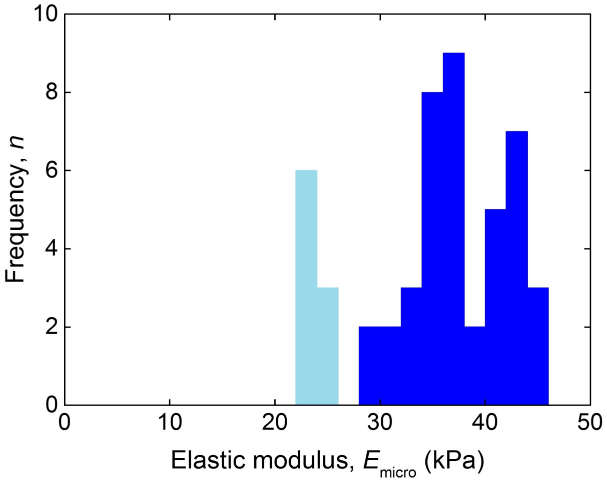

Supplementary Figure S5: Modulus distribution from AFM-based indentation of the gel component of physical cryogels. (**Light blue**) Indentation of water-filled pores, and (**Dark blue**) indentation of the gel component.
